# Psychometric properties of the Hungarian childhood trauma questionnaire short form and its validity in patients with adult attention-deficit hyperactivity disorder or borderline personality disorder

**DOI:** 10.1186/s40479-023-00239-8

**Published:** 2023-11-17

**Authors:** Eszter Kenézlői, Eszter Csernela, Zsófia Nemoda, Krisztina Lakatos, Boldizsár Czéh, Zsolt Szabolcs Unoka, Mária Simon, János M. Réthelyi

**Affiliations:** 1 Doctoral School of Mental Health Sciences, Semmelweis University, Budapest, Hungary; 2 Department of Psychiatry and Psychotherapy, Faculty of Medicine, Semmelweis University, Budapest, Hungary; 3 Department of Psychiatry and Psychotherapy, Medical School, University of Pécs, Pécs, Hungary; 4 Department of Molecular Biology, Institute of Biochemistry and Molecular Biology, Semmelweis University, Budapest, Hungary; 5 Institute of Cognitive Neuroscience and Psychology, HUN-REN Research Centre for Natural Sciences, Budapest, Hungary; 6 Neurobiology of Stress Research Group, Szentágothai János Research Centre, University of Pécs, Pécs, Hungary; 7 Department of Laboratory Medicine, Medical School, University of Pécs, Pécs, Hungary

**Keywords:** Hungarian Childhood Trauma Questionnaire Short Form (H-CTQ-SF), Adult attention-deficit hyperactivity disorder (aADHD), Borderline personality disorder (BPD), Childhood adversity, Early life traumatization, Childhood maltreatment, Principal component analysis, Confirmatory factor analysis, Personality inventory for DSM-5 (PID-5)

## Abstract

**Background:**

Compelling evidence supports the role of childhood traumatization in the etiology of psychiatric disorders, including adult attention-deficit hyperactivity disorder (aADHD) and borderline personality disorder (BPD). The aim of this study was to examine the psychometric properties of the Hungarian version of the Childhood Trauma Questionnaire Short Form (H-CTQ-SF) and to investigate the differences between patients diagnosed with aADHD and BPD in terms of early traumatization.

**Methods:**

Altogether 765 (mean age = 32.8 years, 67.7% women) patients and control subjects were enrolled from different areas of Hungary. Principal component analysis and confirmatory factor analysis were carried out to explore the factor structure of H-CTQ-SF and test the validity of the five-factor structure. Discriminative validity was assessed by comparing clinical and non-clinical samples. Subsequently, aADHD and BPD subgroups were compared with healthy controls to test for the role of early trauma in aADHD without comorbid BPD. Convergent validity was explored by measuring correlations with subscales of the Personality Inventory for DSM-5 (PID-5).

**Results:**

The five scales of the H-CTQ-SF demonstrated adequate internal consistency and reliability values. The five-factor model fitted the Hungarian version well after exclusion of one item from the physical neglect scale because of its cross-loading onto the emotional neglect subscale. The H-CTQ-SF effectively differentiated between the clinical and non-clinical samples. The BPD, but not the aADHD group showed significant differences in each CTQ domain compared with the healthy control group. All CTQ domains, except for physical abuse, demonstrated medium to high correlations with PID-5 emotional lability, anxiousness, separation insecurity, withdrawal, intimacy avoidance, anhedonia, depressivity, suspiciousness, and hostility subscales.

**Conclusions:**

Our study confirmed the psychometric properties of the H-CTQ-SF, an easy-to-administer, non-invasive, ethically sound questionnaire. In aADHD patients without comorbid BPD, low levels of traumatization in every CTQ domain were comparable to those of healthy control individuals. Thus, the increased level of traumatization found in previous studies of aADHD might be associated with the presence of comorbid BPD. Our findings also support the role of emotional neglect, emotional abuse and sexual abuse in the development of BPD.

**Supplementary Information:**

The online version contains supplementary material available at 10.1186/s40479-023-00239-8.

## Background

### Early trauma in the background of psychopathology

Several lines of research suggest that psychopathology emerges as the result of complex interactions between environmental risk factors and genetic vulnerability [1–3]. Among environmental risk factors the most prominent and frequently reported are childhood traumatic experiences, especially abuse and neglect. Across various community samples, exposure to at least one form of abuse in childhood was 26.6% and 31.7% for females and males, respectively [4]. According to a representative survey of the general population in the U.S., the prevalence of sexual abuse was 14.2% for men, and 32.3% for women, while prevalence of physical abuse was 19.5% for women and 22.2% for men [5]. Population-based studies show that 8–25% of children in high-income countries and 10–39% of children in middle-income countries witness interpersonal violence in their homes [6, 7].

Several studies indicate that childhood adversities are associated with a wide range of psychiatric pathologies [8], i.e., mood disorders [9–11], anxiety disorders [12–14], substance use disorder [15, 16], and psychosis [17]. Non-suicidal self-harm [18], suicidal ideation, and suicidal behavior have also been linked with childhood maltreatment in several populations [19, 20]. There is an increasing need for the retrospective detection of early adverse events in order to recognize and prevent the long-term consequences of childhood adversity and maltreatment. It has been suggested that the extreme stress caused by adverse circumstances can affect early brain development, as well as the development of neurohormonal and immune systems [21–23]. In addition to the general effects of stress, maltreatment associated with threats (e.g., physical and sexual abuse) or deprivation (emotional and physical neglect) have profound effects on cognitive and emotional development and subsequent psychopathology [24].

Early life traumatization has also been reported to influence personality development and adult personality structure, affecting several personality domains. Negative affectivity, detachment, and psychoticism have been shown to correlate with early traumatization and mediate between childhood adversities and internalizing symptoms [25]. These findings constitute the foundation for the alternative model for personality disorders (AMPD) in the Diagnostic and Statistical Manual of Mental Disorders 5th edition (DSM-5) Section III [26], and the Personality Inventory for DSM-5 (PID-5) [27, 28].

The etiology of BPD is under the influence of both genetic and environmental factors, being in interaction with each other [29–31]. Distel et al.‘s (2008) twin study found that genetics accounted for 42% of variation in BPD symptoms in both genders across the Netherlands, Belgium, and Australia samples. Environmental influences explained the remaining 58% [32].

Conversely, ADHD has a high heritability of 70–80% [33, 34]. Recent genetic models emphasize the interaction with environmental factors in ADHD as well, including early traumatization. The retrospective study of Rucklidge and co-workers (2006) demonstrated higher prevalence of emotional abuse or emotional neglect in aADHD patients [35]. However, it still is not yet clear whether emotional or physical abuse act as causal factors per se or by mediation of the child’s emotional dysregulation, which creates a challenging experience for the parents. To assess the impact of early trauma on ADHD and BPD, we must consider their high rate of comorbidity. Several prospective studies showed that childhood ADHD was a risk factor for the subsequent development of BPD [36–39], with rates of BPD among adults with ADHD ranging from 19 to 37%. In clinical samples of BPD patients, the prevalence of aADHD is higher than in the general population, ranging from 16 to 38% [40, 41].

In summary, many psychiatric disorders including BPD and aADHD have been associated with childhood maltreatment. Assessing childhood adversity and trauma is essential for both clinical and research settings. To ensure ethical practice and accuracy, reliable assessment tools are needed, which must be able to measure varying degrees of maltreatment severity within different types of trauma. Such tools should be non-intrusive and easily administered [42].

### Measuring early traumatization

Measuring early traumatization is a challenging, yet critical element of clinical evaluation [43], as traumatization in the past influences not only the clinical course of psychiatric disorders (i.e., greater symptom severity), but also treatment response to pharmacotherapy and psychotherapy [44]. There are concerns in the retrospective assessment of childhood maltreatment, e.g., memories can be distorted for several reasons, including the long time lag. In addition, children are usually exposed to only one family environment and do not experience other caregiving circumstances. As a consequence, they consider maltreatment as normal, at least to a certain extent, and it takes time to realize that “things shouldn’t have happened in the way they happened”, and to acknowledge trauma not as a norm. Sensitive characteristics of trauma, e.g., shame, and other negative emotions that accompany these psychological reactions, such as minimization and denial can result in reluctance or inability to communicate problems. There are also ethical and therapeutic concerns about exploring traumas, since they can activate memories and emotional reactions, such as anxiety, flashbacks, and dissociation.

Early traumas can be measured either by self–rated questionnaires or by expert–rated interviews. Questionnaires have the advantage of being economical, easily administered and scored, and assuring anonymity, which might reduce the chance of distorted responses due to shame arising in association with traumas. Retrospective trauma interviews can provide a richer and more detailed description of early traumatic experiences.

The most thoroughly validated, and extensively used instrument to measure the experience of early trauma is the Childhood Trauma Questionnaire Short Form (CTQ-SF; [45]. The CTQ-SF is a retrospective 28-item self-report questionnaire that measures childhood exposure to traumatic experiences in five distinct dimensions: Emotional Abuse (EA), Physical Abuse (PA), Sexual Abuse (SA), Emotional Neglect (EN), and Physical Neglect (PN). By measuring the five types of abuse and neglect, it also takes into consideration the co-occurrence of different types of abusive experiences and individual traumas [45]. It is short and fairly non-invasive, as it asks about the frequency of experiences and events, not their specific details, to maximize the chances of recognizing abuse and neglect. The CTQ-SF has been demonstrated to have good reliability and validity in clinical and community samples [46, 47]. The 5-factor structure of the CTQ-SF has been confirmed in several studies [46, 48–54].

The Early Trauma Inventory (ETI) was created by Bremner et al. (2000) as a comprehensive expert-rated interview [55]. A self-rated version (ETI-SR) was developed subsequently, and a brief self-rated short form was made after a psychometric analysis identified redundant items [56]. ETISR-SF is a valid instrument for retrospective self-assessment of childhood trauma in diverse populations and cultural contexts and has good test-retest reliability. It was translated into several languages while preserving psychometric properties. As it measures not only trauma domains, but also the age of onset, duration and frequency of traumatic events, the perpetrator’s motivations, and the emotional impact of the traumas, it is suitable for use in trauma research and specialized clinical settings [57].

Among other trauma interviews, the Childhood Experience of Care and Abuse (CECA) [58] and the Childhood Trauma Interview (CTI) [59] have received the most empirical attention. Compared to many other trauma interviews, the CECA and the CTI assess a broader range of traumatic childhood events. The CECA has been extensively validated [58, 60, 61], while the validation of the CTI is limited to drug and alcohol use disorder samples [59]. The PID-5 was developed according to the AMPD, and is a personality questionnaire rather than an instrument for the assessment of traumatization per se. However, recent studies reported strong associations between several PID-5 subscales (e.g., anxiousness, depressivity, suspiciousness, hostility, negative affectivity, detachment) and early traumatization [25, 62].

There has been a lack of non-invasive, easy to administer tools with good reliability and validity for detecting childhood adverse events in Hungarian. CTQ was selected for translation and validation due to its advantages discussed above. We also aimed to evaluate the differences between the levels of early traumatization in aADHD and BPD patient groups. To our best knowledge, no previous study measured the level of early traumatization in aADHD after the exclusion of comorbid BPD. We hypothesized that the H-CTQ-SF will be able to discriminate between clinical and non-clinical samples, and subscales will show differences between patients with aADHD and BPD.

## Methods

### Participants

Patients and control subjects were recruited in the capital city of Hungary at Semmelweis University, Department of Psychiatry and Psychotherapy, and a mid-sized town at the University of Pécs, Department of Psychiatry and Psychotherapy, to expand the sample with subjects from rural areas. Both sites provided clinical and non-clinical samples, and were granted ethical approval by relevant research ethics committees. All participants provided written informed consent.

A community sample of 358 subjects without any psychiatric history was recruited in Pécs, in order to test the general feasibility of the H-CTQ-SF and to make some minor changes to improve the translation. This community sample was complemented by 171 psychiatric inpatients recruited in Pécs, who were diagnosed according to DSM-5 criteria. The rates of primary diagnoses in this psychiatric inpatient group were the following: 85.4% mood disorders, 21.2% personality disorders, 15.8% substance use disorders, and 11.7% other. The psychiatric patients enrolled in Budapest were diagnosed with either aADHD (n = 78), or BPD (n = 60) as a main diagnosis. In the aADHD group 53.8%, in the BPD group 93.5% of the subsample had at least one comorbid diagnosis. The secondary diagnoses in the aADHD group were the following: mood disorders 38.5%, substance use disorders 23.1%, anxiety disorders 22%, PTSD 2.2%, obsessive compulsive disorder 1.3%, eating disorder 1.3%, somatic symptom disorder 1.3%, personality disorder other than BPD 11%. In the BPD group comorbidities included mood disorders 82.3%, anxiety 75.8%, personality disorder other than BPD 40.3%, substance use disorder 30.6%, PTSD 27.4%, eating disorder 21%, obsessive compulsive disorder 8.1%, and somatic symptom disorder 4.8%. The screened control group recruited in Budapest consisted of 98 healthy subjects without psychiatric history, who were screened thoroughly by a variety of assessment tools (see below).

Exclusion criteria were the same at both sites: psychosis, neurocognitive or developmental impairment, mental retardation or insufficiency of reading and writing, limiting the abilities of informed consent and assent. Altogether 765 participants were included in the study. Table 1 shows the demographic characteristics of the total sample and each study group.

**Table 1:** Sociodemographic characteristics of the sample, the aADHD, BPD, screened control, psychiatric inpatient groups, and community sample

	Total n = 765	aADHD group n = 78	BPD group n = 60	Screened control group n = 98	Psychiatric inpatient group n = 171	Community sample n = 358	
Mean age (SD) Min Max	32.83 (11.65) 18 75	26.5 (4.59) 18 35	26.2 (4.63) 18 35	26.39 (4.60) 18 35	37.73 (13.94) 18 75	35.20 (12.17) 18 70	
Gender n (%) Male Female Non binary Missing	243 (31.8) 518 (67.7) 2 (0.3) 2 (0.3)	46 (59.0) 32 (41.0) 0 (0) 0 (0)	13 (21.7) 46 (76.7) 1 (1.7) 0 (0)	42 (42.9) 56 (57.1) 0 (0) 0 (0)	60 (35.1) 110 (64.3) 1 (0.6) 0 (0)	82 (22.9) 274 (76.5) 0 (0) 2 (0.6)	
Education n (%) Primary school (8 years) Secondary school (8 + 2 years) Graduation (8 + 4 years) Finished BSc/MSc/PhD Missing	33 (4.3) 45 (5.9) 299 (39.1) 359 (46.9) 29 (3.8)	1(1.3) 1 (1.3) 40 (51.3) 36 (46.2) 0 (0)	5 (8.3) 3 (5.0) 34 (56.7) 18 (30.0) 0 (0)	1 (1) 0 (0) 48 (49.0) 49 (50.0) 0 (0)	22 (12.9) 31 (18.1) 46 (26.9) 43 (25.1) 29 (17.0)	4 (1.1) 10 (2.8) 131 (36.6) 213 (59.5) 0 (0)	

Subjects in the aADHD, the BPD and the screened control group were enrolled at the Budapest site. Subjects in the psychiatric inpatient group and the community sample were recruited in Pécs

### Psychiatric assessment

A board-certified psychiatrist or clinical psychologist interviewed patients of the BPD and aADHD groups at the Budapest site using MINI 5.0 [63] and SCID-5-PD [64] interviews to validate the clinical diagnosis and detect comorbid psychiatric disorders. Comorbid BPD cases detected by the SCID-5-PD were excluded from the aADHD group, while ADHD symptoms detected by the MINI 5.0 resulted in exclusion from the BPD group to ensure the exclusivity of the two main diagnoses, in order to detect the trauma profile in the aADHD group not attributable to comorbid BPD. BPD patients who had no aADHD diagnosis in the past, but met 3 or more attention deficit/hyperactivity symptoms according to the DSM-5 criteria of ADHD, were excluded as well. The screened healthy control group recruited at this site consisted of 98 healthy subjects without any psychiatric history, not using drugs regularly, and screened by Derogatis Symptom Scale (SCL-90) [65] and Conners’ Adult ADHD Rating Scales (CAARS, 66-item version) [66]. To meet inclusion criteria, their Global Severity Index score had to be below 70 (T-score < 70), furthermore, two of the Inattention, Hyperactivity and Impulsivity CAARS domains needed to be below 70.

The Personality Inventory for DSM-5 (PID-5) by APA, 2013 [27, 28] was administered in each group. This questionnaire was developed for the detailed measurement of personality traits in the background of personality disorders following the dimensional approach of personality disorders. Twenty-five personality traits were assessed, creating 5 higher-order domains: negative affectivity, detachment, disinhibition, antagonism, and psychoticism.

The CTQ short form is suitable for assessing five types of abuse and neglect in childhood and adolescence. The questionnaire takes 5–10 min to complete and can be used with clinical and normative subjects, both individually and in groups. It consists of 28 items on five scales: emotional abuse (EA), physical abuse (PA), sexual abuse (SA), emotional neglect (EN), and physical neglect (PN). In the original English version, each scale consists of five items. Three additional items are used to measure the tendency of minimizing or denying abuse, forming the validity subscale. These items are used to detect the denial or underestimation of trauma, and thus reduce this type of bias. The subject evaluates each item on a Likert scale from 1 to 5 based on the frequency of each life event that occurred before the age of 18 years (never = 1, rarely = 2, sometimes = 3, often = 4, very often = 5). The questionnaire also contains reversed items. Therefore, scores on each scale range between 5 and 25.

The items of the validity scale are also evaluated on a Likert scale from 1 to 5, but the scores are evaluated differently: the scale values ​​are converted to binary values ​​(0 and 1). An item score of 1 to 4 is converted to 0, while the value of 5 is re-scored as 1. Therefore, the three items of the validity scale can add up to 0, 1, 2, and 3 points. In the case of a score of 0, the questionnaire results and the completion can be considered valid, while a score of 1 to 3 indicates the likelihood of denial or underestimation and underreporting of maltreatment (false negatives).

This indicator is particularly relevant when the test profile consists of very low trauma scores in most maltreatment areas, a profile suggesting a tendency to pervasively minimize or deny maltreatment. Under these circumstances, the profile of low trauma scores should be interpreted with caution, and other sources of information should be used to verify the absence of abuse and neglect.

The Hungarian version of the instrument was created using the “reverse” method **(Supplementary Methods)**. The original English questionnaire was translated into Hungarian, which was translated to English again by a bilingual professional blind to the original version of the CTQ-SF. The latter translation was compared to the original by two independent researchers not involved in this study and by a research fellow at the Institute of Anglistics, Faculty of Humanities, University of Pécs. Relevant semantic issues were considered and corrected if necessary. Item 10 contains a double negative that is not a common grammatical form in Hungarian. Testing the H-CTQ-SF in a community sample proved that the double negative in item 10 was indeed difficult to understand and compromised the applicability of this item. To rescue item 10, it was re-worded without the double negative.

### Data analysis

SPSS version 27 was used for all statistical analyses, except for confirmatory factor analysis. Internal consistency was calculated using Cronbach’s alpha [67]. Principal component analysis was carried out to explore the factor structure of H-CTQ-SF. Confirmatory factor analysis (CFA) was performed by using the JASP 0.16.1.0 program in order to examine the Hungarian version of the CTQ-SF and its fit with the original five-factor model [46, 48–54]. Since the chi-square test is susceptible to sample size, even a small difference will result in a significant difference as the sample size increases, this study used four fitting indicators: the comparative fit index (CFI), the Tucker-Lewis index (TLI), the root mean square error of approximation (RMSEA), and the standardized root mean square residual (SRMR). The criteria used to evaluate model fit were: CFI and TLI ≥ 0.95, whereas RMSEA and SRMR ≤ 0.05. An advantage of RMSEA is that a confidence interval can be calculated, which provides more information regarding model fit than a point estimate. The upper bound of this confidence interval should be ≤ 0.10 for an acceptable model fit [68]. Discriminative validity was tested by comparing the clinical and non-clinical samples using Mann–Whitney U test, and comparing the BPD, aADHD and screened control groups using ANOVA analyses and Bonferroni post hoc tests. Correlation analyses were carried out between H-CTQ-SF subscales, and separately, between H-CTQ-SF subscales and PID-5 domain scores. The domains of the PID-5 used for correlation analyses were chosen based on clinical relevance, and recent studies [25, 62].

## Results

### Minimization and denial of traumatization

First, we analysed the H-CTQ-SF validity scale. The role of the 3 validity items is to identify responses minimizing or denying abuse and neglect, thus ensuring validity of the analysed data. Of the 765 completed questionnaires 599 were valid, representing 78.3% of the total sample. The number of valid questionnaires was not statistically different in the clinical and non-clinical subgroups (χ² (df = 1, n = 765) = 3.228, p = .072, φ = 0.065). In the clinical sample 252 out of 309 (81.6%), while in the non-clinical sample, 347 out of 456 (76.1%) were considered to be valid. The proportion of the valid questionnaires was highest in the BPD group (96%), which can be due to the fact, that most of the BPD patients were recruited from an inpatient, psychotherapeutic ward, while other patients were recruited at outpatient units, not having a psychotherapeutic profile. In other terms, BPD patients might have been more reflective of their traumas. Only valid questionnaires were used for further analyses.

### Reliability and internal consistency

Internal consistency coefficients for the original CTQ scales, described by Bernstein et al. [46] were computed as Cronbach’s alpha [67] values (Table 2). Reliability coefficients of the H-CTQ-SF scales in the total sample ranged between 0.65 and 0.95 both in the clinical and non-clinical sample, indicating an adequate internal consistency of the H-CTQ-SF.

**Table 2:** Cronbach’s alpha values of the 5 scales of H-CTQ-SF measured in the total valid, clinical and non-clinical sample

Scale – Cronbach’s α	Total n = 599	Clinical sample n = 252	Non-clinical sample n = 347
Emotional abuse (EA)	0.880	0.870	0.874
Physical abuse (PA)	0.873	0.863	0.882
Sexual abuse (SA)	0.934	0.926	0.946
Emotional neglect (EN)	0.876	0.842	0.886
Physical neglect (PN)	0.651	0.636	0.611

### Principal component analysis

Principal component analysis (PCA) was carried out on the total valid sample of 599 cases to explore the factor structure of the Hungarian version of the CTQ-SF and compare it with the original factor structure described by Bernstein et al. [46]. Pair-wise exclusion of cases was used to handle missing values. Since the subscales were known from previous studies to be inter-correlated [45], oblimin rotation was applied. We used Kaiser’s eigenvalues *>* 1; Cattell’s scree test, and parallel analysis using both mean and 95th percentile eigenvalues [69, 70] to determine the number of factors to retain. The five-factor solution accounted for 68% of the variance. Only two cross-loadings were observed in PCA, items 2 and 4 loaded onto the EN, instead of the PN scale (Table 3).

**Table 3:** Results of the principal component analysis (PCA) using the five-factor solution (R² = 0.68) with oblimin rotation and Kaiser normalization

When I was growing up…	EN	SA	PA	PN	EA
13* People in my family looked out for each other.	− 0.785				
28* My family was a source of strength and support.	− 0.784				
19* People in my family felt close to each other.	− 0.778				
5* There was someone in my family who helped me feel that I was important or special.	− 0.726				
7* I felt loved.	− 0.724				
2* I knew that there was someone to take care of me and protect me.	− 0.618				
4 My parents were too drunk or high to take care of the family.	0.432				
20 Someone tried to touch me in a sexual way, or tried to make me touch him/her.		0.963			
24 Someone molested me.		0.941			
27 I believe that I was sexually abused.		0.939			
23 Someone tried to make me do sexual things or watch sexual things.		0.912			
21 Someone threatened to hurt me or tell lies about me unless I did something sexual with them.		0.725			
9 I got hit so hard by someone in my family that I had to see a doctor or go to the hospital.			0.866		
17 I got hit or beaten so badly that it was noticed by someone like a teacher, neighbour, or doctor.			0.835		
11 People in my family hit me so hard that it left me with bruises or marks.			0.811		
12 I was punished with a belt, a board, a cord, or some other hard object.			0.699		
15 I believe that I was physically abused.			0.625		
6 I had to wear dirty clothes.				− 0.822	
1 I didn’t have enough to eat.				− 0.666	
26* There was someone to take me to the doctor if I needed it.				0.611	
3 People in my family called me things like ‘‘stupid,’’ ‘‘lazy,’’ or “ugly”.					0.729
14 People in my family said hurtful or insulting things to me.					0.700
25 I believe I was emotionally abused.					0.598
18 I felt that someone in my family hated me.					0.553
8 I thought that my parents wished I had never been born.					0.422

EN: emotional neglect, SA: sexual abuse, PA: physical abuse, PN: physical neglect, EA: emotional abuse. Loadings below 0.4 are not indicated. * Reversed items

### Confirmatory factor analysis

A confirmatory factor analysis was carried out to assess the structural validity of the H-CTQ-SF in the pooled clinical (aADHD, BPD, psychiatric inpatient group) and non-clinical (screened healthy control group and population sample) groups. The tested five-factor model was based on the factor structure described by Bernstein et al. [46]. Although the original model reached a moderate fit (Table 4), 13 pairs of error variances, that made substantive sense, and loaded on the same scales were freed to covary: items 24 and 27 both refer to sexual abuse, items 13 and 19 refer to “people in my family”, items 3 and 14 are about experiencing hurtful things, items 8 and 18 express hate, items 5 and 7 entail love and importance. Items 9, 11, 12 and 17 refer to severe corporal punishment, thus they were freed pairwise. Items 21 and 23 both refer to the coercion of sexual activities. Item 2 loaded on the EN factor instead of the PN factor, thus it was removed from the items of PN. The goodness-of-fit statistics of the five-factor model with covariation residuals and after removing item 2 proved an excellent model fit (χ^2^ = 648.653, df = 229, p < .001, CFI = 0.956, TLI = 0.947 RMSEA = 0.055, RMSEA CI upper bound = 0.06, and SRMR = 0.044). The explained variances of the items for this model ranged from 0.213 (item 4 on the PN scale) to 0.950 (item 20 on the SA scale).

**Table 4:** Model fit indices of the confirmatory factor analysis models of the H-CTS-SF.

	χ² (df)	CFI	TLI	RMSEA [90% CI]	SRMR
Original 5 factor model	1312.773 (265)	0.896	0.883	0.081[0.077, 0.860]	0.055
5 factor model with reasonable covariation residuals	767.938 (252)	0.949	0.939	0.058 [0.054, 0.063]	0.050
5 factor model without item 2	648.653 (229)	0.956	0.947	0.055 [0.050, 0.060]	0.044

CFI = comparative fit index, TLI = Tucker-Lewis index, RMSEA = root mean square error of approximation, CI = confidence interval; SRMR = standardized root mean square residual

### Correlation analyses

Next, we investigated the intercorrelation of the CTQ subscales in the total valid sample (n = 599). The five scales were in moderate to strong correlation with each other, indicating the co-occurrence of the different traumas, which is consistent with previous studies (Table 5**)**.

**Table 5:** Spearman correlation coefficients between the CTQ subscales

	EA	PA	SA	EN	PN
EA		0.581***	0.347***	0.674***	0.581***
PA			0.305***	0.460***	0.430***
SA				0.252***	0.294***
EN					0.683***

EA: emotional abuse, PA: physical abuse, SA: sexual abuse, EN: emotional neglect, PN: physical neglect,

*** p < .001

We also explored the correlation of H-CTQ-SF subscales with *a priori* selected PID-5 subscales, including emotional lability, anxiousness, separation insecurity, withdrawal, intimacy avoidance, anhedonia, depressivity, suspiciousness, hostility, as these were associated with early trauma in previous studies. The EA, the SA the EN and PN subscales of the H-CTQ-SF showed significant, low to moderate positive correlations in the aADHD, BPD and screened healthy control groups (n = 236), with each analysed subscale of the DSM-5 personality inventory indicating a good convergent validity (Table 6). Values of the PA subscale, albeit positively, were only correlated with the PID-5 anxiousness, separation insecurity, suspiciousness subscales.

**Table 6:** Spearman correlation coefficients between H-CTQ-SF and selected PID-5 subscales in the aADHD, BPD and screened control groups (n = 236)

	Negative affectivity					Detachment				Other traits	
	Emotional lability	Anxiousness	Separation insecurity	Withdrawal		Intimacy avoidance	Anhedonia	Depressivity		Suspiciousness	Hostility
EA	0.295***	0.283***	0.275***	0.222**		0.255***	0.235**	0.293***		0.374***	0.260***
PA	0.084	0.152*	0.157*	0.130		0.093	0.105	0.128		0.246**	0.112
SA	0.221**	0.234**	0.212**	0.218**		0.195**	0.230**	0.259***		0.238**	0.143*
EN	0.232**	0.242**	0.222**	0.227**		0.356***	0.370***	0.341***		0.245**	0.207**
PN	0.173*	0.139	0.182*	0.256***		0.228**	0.248***	0.259***		0.287***	0.159*

EA: emotional abuse, PA: physical abuse, SA: sexual abuse, EN: emotional neglect, PN: physical neglect, * p < .05 ** p < .01 *** p < .001

### Discriminative validity of the H-CTQ-SF

**Fig. 1:**
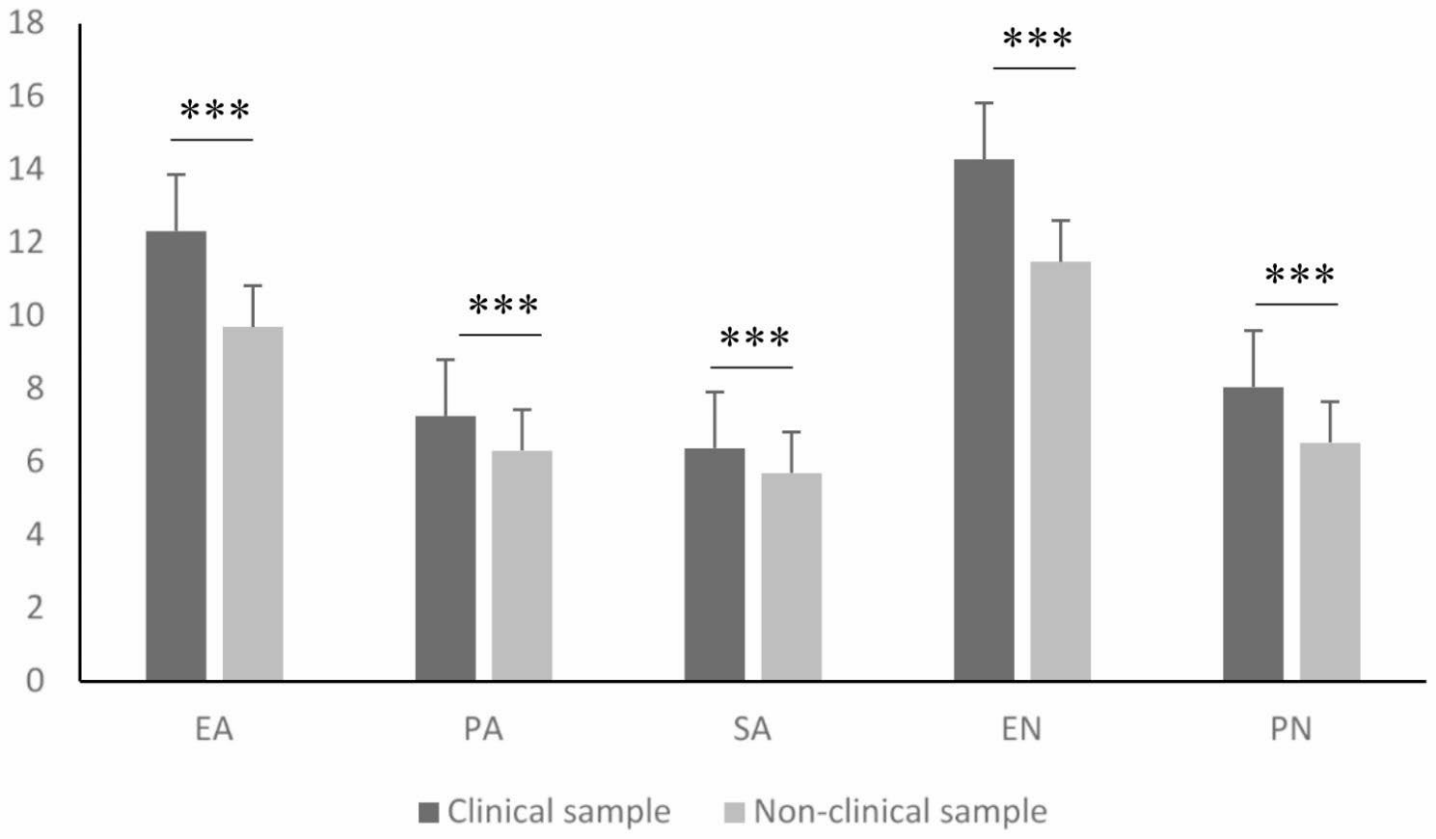
Differences between the clinical (n = 252) and non-clinical (n = 347) samples in terms of CTQ subscales Legend: Values represent mean + standard deviation. Statistics: Mann–Whitney U-test. *** p < .001

Using Mann–Whitney U-test for pairwise comparisons, the clinical and non-clinical samples demonstrated significant differences in each CTQ subscale (Fig. 1).

**Fig. 2:**
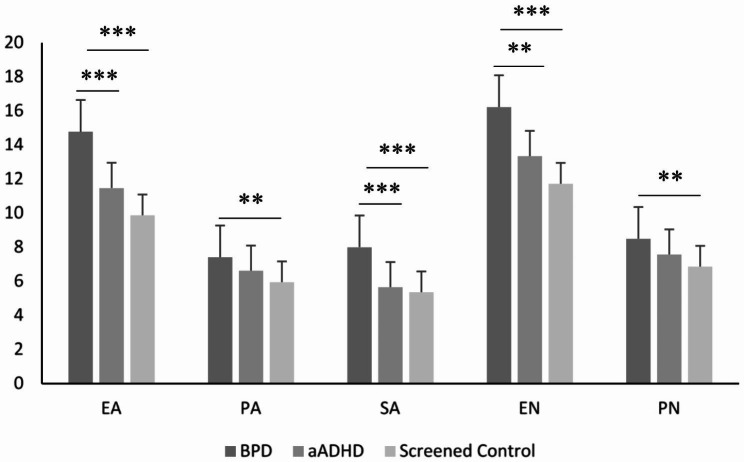
The CTQ subscales in BPD (n = 60), aADHD (n = 78) and screened control groups (n = 98) Legend: Values represent mean + standard deviation. Statistics: one-way ANOVA followed by Bonferroni post hoc tests. ** p < .01; *** p < .001

The sample recruited at the Budapest site consisted of BPD, aADHD and screened control groups, in which subjects with comorbid aADHD and BPD, or healthy control subjects with subclinical symptoms were excluded as a result of the rigorous screening process. Using ANOVA analyses and Bonferroni post hoc tests, the BPD group differed significantly from the control group in each CTQ scale (EA, SA, EN scales p < .001, PA, PN scales p < .01), while there was no significant difference between the aADHD and the screened control group in any of the CTQ subscales. The BPD group had significantly higher values than the aADHD group in the EA, SA (p < .001), and EN (p < .01) scales, but was not different in the PA and PN scales (Fig. 2).

## Discussion

The main findings of this study are the following: (1) The five scales of H-CTQ-SF demonstrated adequate internal consistency and reliability that were similar to the English version of CTQ-SF. (2) The hypothesized five-factor model of the CTQ-SF fitted well with the Hungarian version of the CTQ-SF, even after the removal of one item from the PN scale, due to its cross-loading onto the EN subscale. (3) The H-CTQ-SF effectively differentiated between clinical patients and the population sample, and also between the screened healthy control, aADHD and BPD groups. Early trauma in the aADHD group did not exceed the levels seen in healthy controls. (4) As a convergent validity measure, the H-CTQ-SF showed good correlation with relevant domains of the PID-5 questionnaire.

### Internal consistency and factor structure of the H-CTQ-SF

Cronbach alpha values were satisfactory on the five subscales, ranging between 0.651 (PN) and 0.934 (SA), showing excellent internal consistency of H-CTQ-SF. The PN scale revealed a somewhat lower alpha value, but was well over the limit for acceptable alpha-values (0.50) that can be used for group comparisons. The lower PN alpha value seems to be characteristic not only of the Hungarian version but also of the Chinese, Brazil, Swiss, Spanish, Korean, Dutch, Swedish versions [42, 50–52, 54, 71, 72], and the original English version as well [45]. This could indicate a weakness of the original construction of the PN subscale.

The principal component analysis resulted in a five-factor model that explained 68% of the variance. Only two cross-loadings were observed in PCA, items 2 and 4 loaded onto the EN, instead of the PN scale. These findings may partly be due to the connotation of the phrase “to take care” in the Hungarian translation, which raises more emotional associations. However, in the original study, factor loadings for the items constituting PN were also relatively low, and one subsequent study failed to demonstrate factorial validity of the PN subscale.

Other cultural adaptations of CTQ found similar alterations in the factor structure. This problem may be related to the theoretical inhomogeneity of the “physical neglect” construct. It seems to be relevant to form a PN scale besides the EN scale, similar to the constructions of physical vs. emotional abuse, however Gerdner and Allgulander (2009) suggested other constructs as well [42]. In our sample, items 2 (“I knew that there was someone to take care of me and protect me”) and 4 (“My parents were too drunk or high to take care of the family”) loaded on the EN scale, while in other cultural adaptations item 26 (“There was someone to take me to the doctor if I needed it”) loaded on the EN scale instead of the proposed PN scale. All three items refer to the lack of care, which has both physical and emotional connotations. The two remaining items (1 and 6), with highest loadings on the proposed PN factor, are referring to supply of food and clean clothes. Gerdner and Allgulander (2009) suggested another construct for these items, “lack of supervision”. Based on their former findings, they proposed “neglect of care” and “neglect of supervision” as separate, although correlated factors [42].

In summary, the problematic internal consistency of the PN subscale and the low item loadings onto PN are not indicative of the weakness of the H-CTQ-SF, but instead of inconsistencies in the construct validity of the original version. Additionally, the five-factor structure demonstrated in this study is in line with the findings of the original version, supporting cross-cultural factorial equivalence.

### Discriminative and convergent validity of H-CTQ-SF

In accordance with previous studies, the H-CTQ-SF was able to differentiate between clinical and non-clinical samples. At each trauma domain, the clinical sample showed increased scores and the differences are significant at each CTQ subscale, supporting former findings discussed previously. Each CTQ subscale showed significant positive correlations, from acceptable to moderate, with each analysed subscale of PID-5, indicative of good convergent validity. These results are in line with findings of recent studies in this field showing that early-life traumatization influences personality structure and pathology, which can mediate towards other symptom domains, e.g., dissociation or suicidal behaviour [25, 62].

### The prevalence of early traumatization in the aADHD and BPD groups

The higher level of each trauma domain in BPD and the lack of significant differences between aADHD and the screened control group leads to the assumption that the elevated level of trauma found in former studies of aADHD might be associated with the presence of comorbid BPD, supported also by the results of Rüfenacht [73]. Therefore, the lower level of childhood traumatization found in this study, might not be generalizable to the aADHD population encountered in clinical practice, due to the fact that patients with comorbid BPD were excluded.

According to our results, the environmental factors playing a role in the etiology of aADHD cannot be reliably measured by the CTQ. There was a significant difference between aADHD and BPD groups in the EA, SA and EN subscales, but they did not differ in the PA and PN subscales. A recent review of Calvo (2020) examined the role of early traumatization in the transition of ADHD into adult BPD [74]. Most of the analysed studies describe an increased risk of children with ADHD who report emotional and sexual traumatic experiences to develop BPD in adulthood. Our findings also support the role of emotional neglect, emotional abuse and sexual abuse in the development of BPD.

### Limitations

The following limitations of our study have to be considered: we have not examined patients suffering from both aADHD and BPD, thus, we have no data about the level of early traumatization in this group. Discriminative validity and correlations were only investigated in a subsample. To examine convergent validity instead of another trauma measurement tool, we applied the PID-5 personality questionnaire, which has been shown to correlate closely with trauma measures, however it doesn’t measure early traumatization per se, thus it should be considered a suboptimal surrogate marker.

## Conclusions

In Hungary, no psychological instruments were available to measure adverse childhood experiences in a non-invasive and ethically sound way with good reliability and validity. According to our results, the Hungarian version of the Childhood Trauma Questionnaire Short Form (H-CTQ-SF) has adequate internal consistency and reliability values and effectively differentiates between the clinical and non-clinical samples. Principal component analysis demonstrated that the five-factor model excellently fits the Hungarian version. The H-CTQ-SF effectively discriminated the aADHD and BPD groups, and demonstrated significant correlation with dysfunctional personality traits, measured by PID-5.

Because of its excellent psychometric properties, the H-CTQ-SF can be considered as an important clinical tool that can be used by professionals not only in the field of research, but also in patient-focused psychiatry and psychotherapy.

To our best knowledge, no previous study assessed the level of early traumatization in aADHD cases without comorbid BPD. Early traumatization in this specific aADHD group was significantly lower compared with the BPD group, and was not different compared to the healthy control group. The lack of significant differences between aADHD and the control group in terms of early traumatization leads to the question, whether the elevated level of traumas found previously in aADHD samples might be a consequence of comorbid BPD. Consideration of trauma patterns and the level of childhood adversities is a crucial part of diagnostic and the therapeutic work as well. On one hand, our study fills a gap in terms of measuring the psychometric properties of H-CTQ-SF, an easy-to-administer, non-invasive questionnaire in Hungarian. On the other hand, our findings support the role of emotional abuse, sexual abuse and emotional neglect in the development of BPD, and also provide insight into a much less studied area, the role of traumatization in aADHD cases with and without comorbid BPD.

### Electronic supplementary material

Below is the link to the electronic supplementary material.

Supplementary Material 1

## Data Availability

The datasets used and analysed during the current study are available from the corresponding author on reasonable request.

## List of abbreviations

aADHD: Adult Attention-deficit Hyperactivity Disorder
AMPD: Alternative Model of Personality Disorders
APA: American Psychiatric Association
BPD: Borderline Personality Disorder
CAARS: Conners’ Adult ADHD Rating Scales
CECA: Childhood Experience of Care and Abuse
CFA: Confirmatory Factor Analysis
CFI: Comparative Fit Index
CTI: Childhood Trauma Interview
DSM-5: Diagnostic and Statistical Manual of mental disorders
EA: Emotional Abuse
EN: Emotional Neglect
ETI: Early Trauma Inventory
ETI-SR: Self-Rated version of Early Trauma Inventory
ETI-SR-SF: Self-Rated version of Early Trauma Inventory- Short Form
GSI: Global Severity Index
H-CTQ-SF: Hungarian version of the Childhood Trauma Questionnaire Short Form
MINI 5.0: Mini International Neuropsychiatric Interview 5.0
PA: Physical Abuse
PCA: Principal Component Analysis
PID-5: Personality Inventory for DSM-5
PN: Physical Neglect.
PTSD: Post-Traumatic Stress Disorder
RMSEA: Root Mean Square Error of Approximation
SA: Sexual Abuse.
SCID-5-PD: Structured Clinical Interview for DSM-5 Personality Disorders
SCL-90: Derogatis Symptom Scale
SRMR: Standardized Root Mean square Residual
TLI: Tucker-Lewis Index
