# Supplementary material for: Psychometric properties of the Hungarian childhood trauma questionnaire short form and its validity in patients with adult attention-deficit hyperactivity disorder or borderline personality disorder
Source: Borderline Personal Disord Emot Dysregul. 2023 Nov 17;10:33. doi: 10.1186/s40479-023-00239-8 (PMC10655266; doi:10.1186/s40479-023-00239-8)
Supplement: Supplementary file 1 — Supplementary Material 1 [file 40479_2023_239_MOESM1_ESM.docx]

**Gyermekkori trauma kérdőív^1^**

*(Bernstein & Fink 1998, magyar változat: Kenézlői és mtsai. 2023)*

Milyen gyakran fordult elő gyerekkoromban (=18 éves korom előtt), hogy...

|  |  |  | soha | ritkán | időnként | gyakran | nagyon gyakran |
| --- | --- | --- | --- | --- | --- | --- | --- |
| PN | 1. | Nem kaptam eleget enni. |  |  |  |  |  |
| PNR | 2. | Tudtam, hogy van valaki, aki gondoskodik rólam és megvéd engem. |  |  |  |  |  |
| EA | 3. | Családtagjaim olyan szavakkal illettek, mint „hülye”, „lusta” vagy „csúnya”. |  |  |  |  |  |
| PN | 4. | A szüleim olyan ittasak voltak (vagy drog hatása alatt álltak), hogy nem tudtak gondoskodni a családról. |  |  |  |  |  |
| ENR | 5. | Volt olyan személy a családunkban, aki segített abban, hogy fontosnak és különlegesnek érezzem magam. |  |  |  |  |  |
| PN | 6. | Piszkos ruhában kellett járnom. |  |  |  |  |  |
| ENR | 7. | Éreztem, hogy szeretnek. |  |  |  |  |  |
| EA | 8. | Úgy éreztem, hogy szüleim azt kívánták, jobb lett volna, ha meg sem születek. |  |  |  |  |  |
| PA | 9. | Valamelyik családtagom úgy megvert, hogy orvoshoz kellett vinni vagy kórházba kerültem. |  |  |  |  |  |
| M/D | 10. | Semmit nem akartam volna megváltoztatni a családomban. |  |  |  |  |  |
| PA | 11. | A családtagjaim közül valaki annyira megütött, hogy foltok, horzsolások vagy látható nyomok maradtak utána. |  |  |  |  |  |
| PA | 12. | Szíjjal, pálcával, kötéllel vagy más, kemény tárggyal vertek. |  |  |  |  |  |
| ENR | 13. | A családunkban odafigyeltek egymásra az emberek. |  |  |  |  |  |
| EA | 14. | Családtagjaim sértő vagy bántó dolgokat mondtak nekem. |  |  |  |  |  |
| PA | 15. | Úgy gondolom, hogy engem testileg bántalmaztak. |  |  |  |  |  |
| M/D | 16. | Tökéletes gyerekkorom volt. |  |  |  |  |  |
| PA | 17. | Olyan súlyosan megütöttek vagy megvertek, hogy azt észrevette a tanárom, szomszédunk vagy egy orvos. |  |  |  |  |  |
| EA | 18. | Úgy éreztem, hogy valaki a családból gyűlöl engem. |  |  |  |  |  |
| ENR | 19. | A családunkban közel érezték magukat egymáshoz az emberek. |  |  |  |  |  |
| SA | 20. | Volt, aki megpróbált szexuálisan fogdosni; vagy megpróbált rávenni arra, hogy én fogdossam őt szexuális céllal. |  |  |  |  |  |
| SA | 21. | Valaki megfenyegetett, hogy bántani fog vagy hazugságokat terjeszt rólam, hogyha nem vagyok hajlandó szexuális tevékenységre. |  |  |  |  |  |
| M/D | 22. | A családunk a világ legjobb családja volt. |  |  |  |  |  |
| SA | 23. | Megpróbáltak rávenni szexuális tevékenységre vagy szexuális tartalmú dolgok nézésére. |  |  |  |  |  |
| SA | 24. | Volt, hogy szexuálisan molesztáltak. |  |  |  |  |  |
| EA | 25. | Azt hiszem, hogy engem érzelmileg bántalmaztak. |  |  |  |  |  |
| PNR | 26. | Volt, aki orvoshoz vigyen, ha szükségem volt rá. |  |  |  |  |  |
| SA | 27. | Azt hiszem, engem szexuálisan zaklattak / bántalmaztak. |  |  |  |  |  |
| ENR | 28. | A családom támasz és erő forrása volt számomra. |  |  |  |  |  |

^1^ CTQ-SF – Childhood Trauma Questionnaire – short form
